# Expert consultation using the on-line Delphi method for the revision of syndromic groups compiled from emergency data (SOS Médecins and OSCOUR®) in France

**DOI:** 10.1186/s12889-022-14157-x

**Published:** 2022-09-21

**Authors:** Marie-Michèle Thiam, Leslie Simac, Erica Fougère, Cécile Forgeot, Laure Meurice, Jérôme Naud, Yann Le Strat, Céline Caserio-Schönemann

**Affiliations:** 1 Data Science Division, Santé Publique France, Saint-Maurice, France; 2 Regional Division, Santé Publique France, Saint-Maurice, France

**Keywords:** Delphi, Syndromic group, Expert consensus, Syndromic surveillance

## Abstract

**Background:**

Consultation data from emergency general practitioners known as SOS Médecins and emergency departments (ED) from OSCOUR® network to the French syndromic surveillance system SurSaUD® (Surveillance sanitaire des urgences et décès). These data are aggregated and monitored on a daily basis through groupings of one or more medical symptoms or diagnoses (“syndromic groups” (SG)).

The objective of this study was to evaluate, revise and enrich the composition of SGs through a consensus of experts who contributed or have experience in syndromic surveillance.

**Methods:**

Three rounds of a Delphi survey were organised, involving 15 volunteers from SOS Médecins and 64 ED physicians in the OSCOUR® network as well as 8 international epidemiologists. Thirty-four SOS Médecins and 40 OSCOUR® SGs covering major medical specialities were put to the experts, along with their diagnostic codes and their surveillance objectives. In each round, the experts could retain or reject the codes according to the surveillance objective. The panel could also put forward new diagnostic codes in the 1st round, included in subsequent rounds. Consensus was reached for a code if 80% of participants had chosen to keep it, or less than 20% to reject it.

**Results:**

A total of 12 SOS Médecins doctors (80%), 30 ED doctors (47%) and 4 international experts (50%) participated in the three rounds. All of the SGs presented to the panel included 102 initial diagnostic codes and 73 additional codes for SOS Médecins, 272 initial diagnostic codes and 204 additional codes for OSCOUR®. At the end of the 3 rounds, 14 SOS Médecins (40%) and 11 OSCOUR® (28%) SGs achieved a consensus to maintain all of their diagnostic codes. Among these, indicators of winter seasonal surveillance (bronchiolitis and gastroenteritis) were included.

**Conclusion:**

This study involved a panel of national experts with international representation and a good level of involvement throughout the survey. In the absence of a standard definition, the Delphi method has been shown to be useful in defining and validating syndromic surveillance indicators.

**Supplementary Information:**

The online version contains supplementary material available at 10.1186/s12889-022-14157-x.

## Introduction

Syndromic surveillance is defined as the collection, analysis, interpretation and dissemination of health data in real-time or close to real-time, in the aim of early identification of an impact (or lack of impact), a potential threat to human or animal health, the management of which may require the implementation of public health actions [1]. Unlike most pathogen-centric epidemiological surveillance systems based on laboratory analysis results, syndromic surveillance is based on the collection of clinical data (symptoms and/or medical diagnoses). The data collected are produced by health professionals as part of their routine activity, for their own needs and from their work repository (operating software, medical thesaurus, coding practices) and not for *a theoretically* defined health surveillance objective, which must be taken into account when compiling indicators or analysing them. The ability of the system to detect an epidemic or a public health threat earlier is highly dependent on the upstream definition of indicators as well as on the choice of sensitivity or specificity criteria to group patients according to coherent diagnostic groups [2, 3]. In general, surveillance is reactive, more often sensitive than specific and completes the information available through other surveillance systems, particularly specific systems [1].

In France, the SurSaUD® syndromic surveillance system (Surveillance Sanitaire des Urgences et des Décès) was developed in 2004 following the health crisis stemming from the exceptional heat wave of 2003 [4, 5]. It is led by Santé publique France (SpFrance), the French Public Health Agency, in the aim of detecting unexpected or known health events early on, monitoring seasonal epidemics, quickly assessing the impact of an event on the recourse to emergency care (morbidity) and/or mortality [4]. With 17 years of past data, the system is increasingly used to conduct studies in order to document a trend or assess the burden of a specific health event [4].

In SurSaUD®, the indicators analysed daily are syndromic groups (SG) consisting of one or more symptoms and/or diagnoses made following a medical examination, without systematic and/or immediate biological confirmation (especially for SGs used for surveillance and alert purposes). They were compiled by epidemiologists as soon as the system was set up, to meet different surveillance objectives. They have varying characteristics of sensitivity or specificity according to these objectives [6]. These groups have been worked on several times: creation, modification according to changes in the analysis strategy or after discussion with the professionals of the data provider networks (definition and dissemination of coding recommendations) [6]. However, their content has never undergone systematic validation or scientific evaluation for all indicators [7, 8].

Several methods can be used to build SGs: experience from previous epidemics, use of medical literature, individual consultation with clinicians. However, an absence of gold standard for many indicators (other than surveillance of infectious diseases covered by conventional systems) in addition to a lack of resources restrict the use of these approaches [7, 8]. Consultation with experts using the Delphi method was chosen because of the ease of submission of an on-line questionnaire to a large number of people within a relatively short survey period. It is used in a variety of areas including that of health: mental health research [9], choice of quality of care indicators [10], evaluation of health promotion programs [11], definition of epidemic periods of influenza [12]. Furthermore, this method complies with scientific rules which makes it reproducible and applicable to other systems.

The primary objective of this study was to evaluate or revise the composition of around 40 SGs, monitored daily, based on expert consensus, in order to validate them and to improve consistency between surveillance requirements and clinical practices.

## Material and methods

### The French syndromic surveillance system SurSaUD®

SurSaUD® collects morbidity data through OSCOUR® networks (Organisation for the coordinated surveillance of emergency admissions) and SOS Médecins. Each day in 2019, nearly 700 ED (93.3% national coverage, 51,000 ED visits per day on average) and 62 out of 63 SOS Médecins associations (98.4% coverage, daily 10,880 medical procedures on average) automatically send to Santé publique France their activity data [4]. For each visit, the following standardised information is available: demographic data (date of birth, gender, post code and city of residence), administrative data (date and time of admission, origin, post-visit referral (hospitalisation, return home)), reasons for consultation and medical diagnoses.

Almost 74% of these ED visits include at least 1 medical diagnosis (80% visits at day+ 3), and several medical diagnoses can be entered. These diagnoses are coded using the International Classification of Diseases, 10th revision (ICD-10), which currently contains almost 40,000 codes and extensions.

On average, 95% of the SOS Médecins data included at least 1 to 3 medical diagnoses coded with 2 specific SOS Médecins thesauruses, which contain a little over 600 different codes.

### Compilation of syndromic groups in SurSaUD®

SGs are compiled specifically for each network. They are based indiscriminately on the main medical diagnosis or secondary medical diagnoses. Diseases and surveillance objectives are convergent between the two sources but the composition is different due to individual thesauruses and medical practices.

In SurSaUD®, among a hundred SGs, 34 SGs were selected for SOS Médecins and 40 SGs for OSCOUR® to be submitted in the current survey. For each, the surveillance objective is specified. It had to be sensitive to detect the maximum use associated with a health event, whether at the stage of suggestive symptoms or declared disease with a (forecast) clinical diagnosis or confirmed by additional tests. However, it had to be specific to monitor in particular the use of a given health event in order to document a trend over time or space or to measure the burden thereof.

### Organisation of the Delphi survey

#### Description of the method

The Delphi method is a group effort that gathers and summarises the knowledge of anonymous, geographically-distant participants who never meet during the survey [13].

The experts are questioned several times on the same questionnaire in successive rounds. Before each round, they receive the results obtained in the previous round in order to compare their opinion with that of the other participants. They can thus modify their responses or not with regard to those of the group [13].

#### Recruitment of experts

Doctors were recruited from the 2 networks along with international participants, between September and December 2018.

The SOS Médecins associations national coordination office put forward a list of 15 doctors in 14 associations. They all confirmed that they wished to participate in the survey.

After contacting them and information being provided to them by the French Society of Emergency Medicine (SFMU), by the members of the steering committee of the OSCOUR® network and by the Fédération nationale des Observatoires des urgences (FEDORU), 64 ED doctors freely agreed to participate.

Internationally, 36 US syndromic surveillance experts from the Centers for Disease Control (CDC) and Prevention’s National Syndromic Surveillance Program (NSSP) were contacted. Eight of them signed up for the survey. Their participation made it possible to envisage comparability for international health risks.

All contact persons who wished to participate were provided with a copy of the survey protocol that included a summary description and the survey objectives and process.

#### Development of the on-line questionnaire

For all three rounds, given the thesaurus in both networks, two questionnaire models were developed using the Limesurvey on-line questionnaire development tool: one for French and American participants (medical diagnoses with ICD-10 codes) and one for SOS Médecins doctors (specific thesaurus). A reminder of the objectives and instructions of the survey were sent in the invitation e-mail and posted on the first page of the questionnaire. Each participant had the opportunity to decline the invitation or not. Due to the large number of SGs to be evaluated and the willing to limit response times and thus ensure better adherence of participants throughout the survey, different sets of SGs were assigned to 2 subgroups for SOS Médecins and 4 subgroups for ED physicians and international experts; the subgroup 3 of SOS Médecins were assigned the overall 34 SGs. The SGs were grouped by theme-based chapter. In the various surveys, 102 diagnostic codes for SOS Médecins and 272 diagnostic codes (and 3000 extensions) for OSCOUR® were submitted. The average number of diagnostic codes per SG was 4.3 [1 (bronchiolitis); 21 (ear, nose, throat (ENT) infection)] for SOS Médecins and 11.4 [1 (bronchiolitis); 40 (fever and rash)] for OSCOUR®.

For the first round questionnaire, the participant was asked to give their opinion on the composition of each SG. The proposed medical diagnostic codes could be selected or not, depending on their relevance to meet the set surveillance objective. This selection was to be based on their clinical expertise, regardless of the software tools available at their own facility. In the first round only, the respondent could suggest codes to be added to the SG for it to meet the surveillance objective in the best possible manner. To help them put forward other codes, SOS Médecins participants received a copy of the specific thesaurus and the ED doctors received an Internet link to the ICD-10 thesaurus (while US participants received a file from the ICD-10-2018 in English, downloaded from the CDC website; [14, 15]). For SGs with many diagnostic codes using ICD-10, preliminary work was carried out to minimise the number of codes to be displayed and to facilitate readability of the questionnaire content.

For the second and third questionnaires, the results of the previous rounds were displayed as a bar chart. Each bar represented the number of times a given code was ticked relative to the total number of participants (expressed as a percentage) (Additional file 1). The respondent was thus able to keep their choice or modify it according to the results of the previous rounds. Only those diagnostic codes that did not reach consensus and those suggested in addition to the first round (marked with an asterisk) were (re)submitted in subsequent rounds.

#### Survey process

A joint pilot survey was organised involving 4 doctors from each network. Its objective was to test the questionnaire but also to complete the SGs with additional diagnostic codes before they were submitted to the various groups.

The survey with SOS Médecins took place from January to May 2019 and the survey with OSCOUR® from April to December 2019. The participants had 15 days to complete the questionnaire after it was sent. At the end of this period, a reminder was sent to participants who did not respond and did not decline the invitation, giving them a further 2 weeks to respond. Between rounds, a period of 1 month was necessary to analyse the responses of the participants in the previous round and prepare the questionnaire for the next round.

Thus, the survey protocol contained different points to ensure validity and reliability of the output. First, although the various networks of physicians suggested list of persons to contact, participation remained voluntary and no compensation was provided for in this survey. The persons to contact have to be located in various ED in the whole French territory and the protocol also planned to include international participants, in order to ensure reliability of results. Furthermore, the survey was anonymous and the results of each round were presented to participants such as it could not be possible to identify the responses from anyone. During the survey, the regular reminders sent to participants who did not respond contributed to maintain participation during the different steps of the study. Finally, in order to reduce bias due to possible variable knowledge of diagnosis thesaurus by participants, a list of additional codes were provided to all participants to help them to put forward other codes into SG during the first round.

### Consensus measurement

Consensus was reached for a diagnostic code if at least 80% of the participants selected it, resulting in inclusion in the SG. If less than 20% of participants chose a code, then it was excluded from the SG. Diagnostic codes that did not reach consensus were submitted in the next round for validation. This consensus assessment approach was explained to the experts in the invitation letter.

## Results

### Participation

The survey of the SOS Médecins network took place with an average interval of 6 weeks between rounds. Twelve out of 15 doctors from SOS Médecins associations took part in the three rounds with an 80% participation rate in the 1st round (*n* = 12), 100% in the 2nd and 3rd rounds (Additional file 2a and b).

The average time between two rounds of the OSCOUR® survey was 8 weeks with a two-month break during the 2019 summer holidays. In total, 30 ED doctors out of 64 (47%) and 4 international experts out of 8 (50%) responded to the three rounds of the survey with a participation rate of 66% (*n* = 42) and 63% (*n* = 5) in the first round, 86% (*n* = 36) and 80% (*n* = 4) in the second round, 83% (*n* = 30) and 100% (*n* = 4) in the third round (Additional file 2a and b).

In the 1st round, all SOS Médecins respondents (100%), 27 ED doctors (64%) and 1 international participant (20%) said they were aware of the SurSaUD® syndromic surveillance system. Eleven out of 12 (92%) SOS Médecins and 33 out of 42 (79%) OSCOUR® respondents reported they had more than 10 years’ medical professional experience. International participants in the OSCOUR® survey were all experts in syndromic surveillance.

For the SOS Médecins network, participants came from 11 of the 13 French regions, with 1 to 2 respondents for each. For the Oscour® network, they came from 9 of the 13 French regions with 1 to 9 per region.

### Syndromic groups

Thirty-four SGs (including 24 with a sensitive objective and 10 with a specific objective) for SOS Médecins and 40 SGs (31 with a sensitive objective and 09 with a specific objective) for OSCOUR® were submitted during the various rounds. Some of them were only present in one of the 2 surveys, while 25 were common to the OSCOUR® and SOS Médecins surveys (Table 1). These SGs covered both general and toxicological signs and organ specialities: Cardiology, Chest medicine, Gastroenterology, Neurology, Psychiatry, Dermatology, Traumatology and Urology.

**Table 1:** Syndromic groups submitted in the Delphi survey by speciality and according to the surveillance objective (sensitive or specific) and the network of partners

	OSCOUR® and SOS Médecins		SOS Médecins		OSCOUR®	
	Sensitive (***n*** = 19)	Specific (***n*** = 6)	Sensitive (***n*** = 5)	Specific (***n*** = 4)	Sensitive (***n*** = 12)	Specific (***n*** = 3)
**General signs**	Impaired general condition Fainting (not including heart disease) Isolate fever Hyperthermia and heat stroke Dehydration		Conjunctivitis		Hyponatraemia	–
**Toxic**	Alcohol Toxic effect of arthropods (including insect bites)	–	–	–	Animal toxic effect (not including arthropods) Toxic effect of plants	–
**Cardiology**	Arrhythmia	–	Heart failure Myocardial ischemia	–	Chest pain State of shock	–
**Respiratory**	ENT infection Acute bronchitis Pneumopathy Acute lower respiratory tract infection Acute respiratory failure	Asthma Bronchiolitis Influenza, flu-like syndrome	–	–	Dyspnea	Chronic respiratory failure
**Digestive**	Vomiting Diarrhoea Acute abdominal pain	Gastroenteritis			–	–
**Neurology**	Headache/migraine Acute confusion		Stroke	Meningitis	Seizure Vertigo Coma	–
**Psychiatry**		Dementia	Anxiety		Anxiety disorders Stress	–
**Dermatology**	Burns and corrosion			Scabies	Skin rash	Effects of solar UV
**Injuries**	–	injuries			–	Drowning
**Urology**	–	–		Renal colic Urinary tract infection	–	

For SOS Médecins, 73 diagnostic codes in 25 SGs were suggested by participants, whereas there were 204 codes for OSCOUR®, divided among 32 SGs. For example, coma and seizure SGs in OSCOUR® initially included 6 codes each. At the end of the survey, the 28 suggested codes for coma and 9 out of the 12 seizure codes reached a consensus to be kept.

### Consensus

At the end of the three rounds, 14 SOS Médecins (44%) and 11 OSCOUR® (28%) SGs reached consensus for all their codes (inclusion or exclusion). Their surveillance objective, their composition and the results produced in Delphi can be found in Additional file 3a and b. Winter surveillance indicators included bronchiolitis and gastroenteritis. The latter had a specific surveillance objective and initially included 1 to 3 diagnostic codes, all kept at the end of the survey.

By broadening the scope to the group of SGs that reached a consensus for 75 to 99% of their codes, 17 SOS Médecins (+ 3) and 27 OSCOUR® (+ 16) SGs were found, bringing SGs with at least 3/4 of their codes retained, including 9 SGs common to both networks, to 50 and 68%, respectively. In addition to monitoring indicators for winter epidemics, SGs used for summer surveillance (hyperthermia/heat stroke, insect bites/arthropod toxic effects), as well as injuries, abdominal pain, headache/migraine, and burns/corrosion, were also identified among the joint SGs (Table 2).

**Table 2:** Breakdown of syndromic groups according to their proportion of diagnostic codes that reached consensus to be kept or rejected, per survey

Proportion of diagnostic codes with consensus	SOS Médecins	OSCOUR®
100%	***n*** **= 14**	***n*** **= 11**
	Meningitis Bronchiolitis Influenza, flu-like syndrome Gastroenteritis Vomiting Diarrhoea Abdominal pain Acute confusion Burns and corrosion Hyperthermia and heat stroke Conjunctivitis Trauma Scabies Urinary tract infection	Animal toxic effect Drowning Seizures Coma Arrhythmia Chest pain Asthma Bronchiolitis Acute bronchitis Gastroenteritis Hyponatraemia
75-99%	***n*** **= 3**	***n*** **= 16**
	Acute respiratory failure, Insect bite, Headaches/migraines	Alcohol Effects of solar UV Burns and corrosion Arthropod toxic effect Plant toxic effect Abdominal pain Headaches, migraines Isolated fever Dementia Injuries Stress ENT infection Dizziness Hyperthermia and heat stroke Skin rash Influenza, flu-like syndrome
50-74%	***n*** **= 14**	***n*** **= 8**
	Impaired general condition Asthma Acute lower respiratory tract infection Stroke Alcohol Dehydration Heart failure ENT infection Myocardial ischemia Acute bronchitis Pneumopathy Isolated fever Arrhythmia Renal colic	Anxiety disorders Dyspnoea Acute respiratory failure Impaired general condition^a^ Vomiting Acute confusion Dehydration Pneumopathy
< 50%	***n*** **= 3**	***n*** **= 5**
	Dementia Anxiety Fainting	Chronic respiratory failure State of shock Acute lower respiratory tract infection Diarrhoea Fainting

^a^At least 50% of the codes came to a consensus for rejection

## Discussion

The Delphi survey collected expert opinions on the contents of SGs created more than 10 years ago to meet the surveillance objectives of SurSaUD®. If consensus was found for only part of the submitted SGs, codes proposed by the participants were added to others.

### Selection and participation of experts

SGs are indicators based on clinicians’ consultations and perceptions. It was therefore important to link the surveillance objectives with coding practices by clinicians, hence their inclusion in the indicator review process as they provide the medical expertise useful to the definition of SGs [8]. Almost all the SOS Médecins survey experts and just over half of the experts in the OSCOUR® survey said they knew about the SurSaUD® system. This knowledge of the SurSaUD® system may have contributed to a proper understanding of the surveillance objectives by the experts and influenced their opinion in choosing the composition of SGs. However, it is difficult to assess the (positive or negative) impact on the survey.

This knowledge of the network also may have helped to maintain a high participation rate as of the first round, especially for the SOS Médecins survey, but also in the 2nd and 3rd rounds, despite the longer time frames than initially planned. Among the volunteers initially enrolled in the survey, the participation of more than 3 out of 4 experts in the SOS Médecins survey and 1 expert out of 2 in the OSCOUR® survey was recorded. Among the respondents in the 1st round, a participation rate of more than 80% in the 2nd and 3rd rounds for the 2 surveys was observed.

The survey was anonymous and the results of each round could not be used to identify the responses from anyone, so as not to influence respondents in their future choices.

### Survey process

The survey took place in three separate rounds over a period of 5 months for SOS Médecins and 9 months for OSCOUR® in 2019. The number of codes to be submitted in each survey was very different, due to the difference in the thesauruses, in their content and the number of codes available. The OSCOUR® SG survey involved too many codes and subcodes, requiring discussion on their display upstream, to facilitate reading and understanding of the survey, while optimising the time needed to respond to them. To do this, developments that were not initially planned were necessary in order to allow user-friendly display of subcodes in tooltips by rolling over diagnostic codes (display method used again to return the results after each round). This approach probably had a positive impact on maintaining the participation rate over the course of the survey. In addition, the OSCOUR® survey was interrupted during the summer holidays (2 months in total), as some areas are impacted by an increase in their tourism-related activity, leaving only a little time to respond to this type of survey for the ED physicians involved. Extending the duration of the survey had negative impacts, such as the higher number of reminders for SOS Médecins only. This may also have led to a memory bias of the participants, even if it was partially made up for by use of the bar chart corresponding to the response selected in the previous round.

### Delphi method for compiling syndromic surveillance indicators

Although syndromic surveillance has existed for several years and is widely used [16, 17], there is no reference definition for SGs, which would otherwise make it possible to facilitate the exchange or comparison of data between systems and to evaluate performance [8].

To our knowledge, this is the first time that the Delphi method is used to work on the definition and composition of SGs. In existing publications, the method often used is that of a group consensus reached after a discussion meeting [7, 8]. Using the Delphi on-line method, a panel of experts working in different geographical areas could be consulted without needing to schedule or travel to any meetings. In addition, given the large number of SGs to be reviewed, several discussion sessions would have been required to reach a result for all SGs. This would likely have been a barrier to the participation of several clinical and international experts, and their workload would not allow them to be as closely involved in this type of project.

Finally, this approach also measured a consensus percentage, which was a more objective decision-making aid for the codes to be maintained or not in each SG.

### Consensus level reached

The SGs for which consensus on codes was reached as early as the first round had a specific surveillance objective.

In syndromic surveillance, sensitivity is used to detect the highest number of patients likely to be in the early stage of a disease that is not yet characterized (with presentation of little specific signs) while specificity is used to refine investigations if a large number of cases with similar symptoms are identified [8]. In studies conducted on the performance of SGs, a better positive predictive value is observed when the surveillance objective is specific rather than sensitive [7, 8].

The diagnostic codes for SGs used for winter surveillance (bronchiolitis, gastroenteritis) were all kept from the 1st round. The specific objective and the small number of codes they comprised probably facilitated consensus among the experts. Used every season for many years, they are used in region-based, multi-source surveillance, which is widely communicated, with weekly reports published and meetings held with data provider partners, during which they were regularly reworked. This visibility can also help healthcare professionals see how seasonal surveillance can make sense, as it is carried out in the aim of contributing to the organisation and adaptation of the care offer, directly benefiting clinicians in their daily activity. These hypotheses could also explain the results for hyperthermia/heat stroke or insect bite SGs, traditionally used in summer surveillance, even if both SGs have poorer consensus, especially the latter, or for trauma and abdominal pain SGs, which correspond to the diagnoses found most frequently in the lead among the top 10 reasons for recourse to emergency medicine.

There is difficulty in reaching consensus for SGs with a sensitive objective that more often incorporates symptoms (diarrhoea, abdominal pain, anxiety disorders, stress, etc.). Among them, some codes of impaired general condition SG (OSCOUR®) were rejected while others did’nt reach consensus. This SG reflects a clinical picture that can have variable aetiologies and come with several pathologies, which means that it may be perceived differently from one clinician to another. This example showed that the surveillance objectives were not always sufficiently explicit or the responses of experts in line with the expectations of epidemiologists. Thus, the participants’ responses focused on supporting the end of life or the condition of the elderly, whereas, for epidemiologists in charge of health surveillance, this SG aimed to measure the sudden deterioration in a patient’s condition (with or without clearly identified aetiology).

Another example is lower respiratory infection SG for which most of the codes did not reach consensus. However, this SG relates to several issues, particularly in the surveillance of winter respiratory diseases [18].

SGs with a sensitive objective are composed of a multitude of diagnostic codes that can be a barrier to reaching a consensus. In addition, as not all codes and subcodes were displayed, this certainly influenced participants’ choice and could partly explain the lack of consensus for some codes. It is not certain that all participants saw the tool tip displayed on rolling over certain codes with the mouse, thus meaning they only read part of the subcodes in the selection in response to the set surveillance objectives.

There was little discrepancy between the responses of international experts and those of the ED specialists. Despite the lack of a reference definition, these results suggest that the development of indicators coincides between countries and thus allows for the comparison of observations between international systems, which is a strong point in the case of international threats.

More generally, the outputs highlighted two limits of using the complete ICD10 classification (40,000 codes) to code medical diagnosis in ED. First, this classification contains symptoms which should not be used for coding medical diagnosis [19]. This symptoms would be more relevant for coding chief complaint. These codes of symptoms in the definition of SG may partly explain why consensus was not reached for these SG. Furthermore, it had been showed that a unconstrained data sets with a large number of codes available for ED give poor data [20]. The usability of a system is an important factor in the quality of data we collect [21]. Based on this observations, a study was conducted by ED syndromic system in UK. With a panel of expert, they proposed a limited list of about 1200 codes based on SNOMED ontology, without any symptoms in this list [22].

In France, a similar process was launched to revised the format for collecting ED data. Particularly, a new format for ED data proposed three major evolutions: for coding medical diagnosis, a list of 1500 ICD10 codes were defined, instead of the entire ICD10 classification. Symptoms codes are removed from this list. A thesaurus were also proposed for coding chief complaint (in the current format, information were collected in free text, without thesaurus). Finally an additional information would be collected to indicate circumstance of the ED visit. This new format is still on discussion and may be implemented soon.

### Review of SGs and implications for epidemiological surveillance

Even if the survey made it possible to add diagnostic codes initially absent and proposed by the experts to SGs, other diagnostic codes were not selected and the differences should therefore be discussed with the expert group.

The epidemiological impact of the results of this survey on the composition of SGs should be analysed by comparing the temporal dynamics of the former and the new composition for each SG. It would also be relevant to assess the performance of SGs by calculating their sensitivity and specificity with regard to the diagnoses actually mentioned in the patient records, however, such studies at national level are burdensome and expensive and can only be considered over a small scope, be it geographic or in terms of the SG selected.

## Conclusion

The definition of SGs is a key element of syndromic surveillance. It must be evaluated on a regular basis and is subject to change depending on surveillance requirements and to be consistent with the set objectives. The Delphi method, by consultation with experts, may be used to validate the contents of SGs with regard to the objectives and systems concerned. The results of this survey are applicable to any other syndromic surveillance system using emergency medicine data, at national or local levels, depending on the organization of surveillance system. The planification of the survey have to be done upstream and take into account many factors like: the availability of experts along the process, the period of the year (with special attention to the vacation periods), the tool to be used (cost of the license, right of use, adaptations needed), the human resources dedicated to the survey, extra time for unexpected events.

However, for SGs with several diagnostic codes that did not reach a consensus at the end of the three rounds, discussions with the experts will be necessary to fully understand the reasons for lack of consensus, the determinants of the various choices. Effectively understanding this will prove useful when compiling the next SGs and more broadly for the organization of a new Delphi survey.

## Supplementary Information

**Additional file 1.** Screenshot of the OSCOUR® survey questionnaire for Injury SG in the 2nd round. This figure shows the display of the questionnaire for the syndromic group “injury” in the 2nd round of the Delphi OSCOUR® survey. On this screenshot, we have at the top the remind of surveillance objective. On the graph, each bar represents one diagnostic code, the proportion of consensus is represented on the abcissa axis. The blue color on the bar indicates to the participant the code he chose while the grey color indicates the codes he did not choose. Below the graph are listed the ICD-10 codes of syndromic group “injury”, the participants were invited to indicate again relevant diagnostic codes according to the surveillance objective. By rolling over T79 with the mouse, they could view included subcodes in the dark blue box.

**Additional file 2.** a. Number of participants contacted and having answered the Delphi SOS Médecins survey. The first column indicates the different rounds of the Delphi SOS Médecins survey. The next 3 columns indicate results for the different groups of the survey. The last column shows the total for all the three groups. The 3 first lines give the number of people per group to whom the questionnaire was sent for each round. The last 3 rows show the number of people who responded to the questionnaire and the participation rate in percentage (number of persons who completed the questionnaire by the number of persons contacted) in each group, for each round. b. Number of participants contacted and having answered the Delphi OSCOUR® survey. The first column indicates the different rounds of the Delphi OSCOUR® survey. The next 3 columns indicate results for the different groups of the survey. The last column shows the total for all the three groups. The three first line give the number of people per group to whom the questionnaire was sent for each round. The last 3 rows show the number of people who responded to the questionnaire and the participation rate in percentage (number of persons who completed the questionnaire by the number of persons contacted) in each group, for each round.

**Additional file 3.** a. Diagnostic codes by syndromic groups (SG) (*n* = 14) and their proportion of consensus in the 3 rounds of the Delphi SOS Médecins survey. The first column indicates the syndromic group, the 2nd column the surveillance objective and the 3rd the label of diagnostic codes. Proportions of consensus are indicated in column 4th to 6th. And the last column indicates if the diagnosis was kept or no in the syndromic group. b. Diagnostic codes by syndromic groups (SG) (*n* = 11) and their proportion of consensus in the 3 rounds of the Delphi OSCOUR® survey. The first column indicates the syndromic group, the 2nd column the surveillance objective and the 3rd the label of diagnostic codes. Proportions of consensus are indicated in column 4th to 6th. The last two columns indicate if the diagnosis was kept or no in the syndromic group and the number of subcodes.

## Data Availability

All data generated or analysed during this study are included in this published article [and its supplementary information files]. They are presented as aggregated results. The datasets used and/or analysed during the current study are available from the corresponding author on reasonable request.

## References

[1] Ziemann A (2011). Assessment of syndromic surveillance in Europe. Lancet.

[2] Henning KJ (2004). What is syndromic surveillance?. MMWR Suppl.

[3] Mandl KD, Overhage JM, Wagner MM, Lober WB, Sebastiani P, Mostashari F (2004). Implementing syndromic surveillance: a practical guide informed by the early experience. J Am Med Inform Assoc.

[4] Caserio Schonemann C, Bousquet V, Fouillet A, Henry V (2014). Le système de surveillance syndromique SurSaUD®. Numéro thématique. La surveillance syndromique en France en 2014. Bull Epidemiol Hebd.

[5] Caserio-Schönemann C, Meynard JB (2015). Ten years experience of syndromic surveillance for civil and military public health, France, 2004-2014. Eurosurveillance.

[6] Fouillet A, Franke F, Bousquet V, Durand C, Henry V, Golliot F (2014). Principe du traitement des données du système de surveillance syndromique SurSaUD® : indicateurs et méthodes d'analyse statistique. Numéro thématique. La surveillance syndromique en France en 2014. Bull Epidemiol Hebd.

[7] Bouchouar E, Hetman BM, Hanley B (2021). Development and validation of an automated emergency department-based syndromic surveillance system to enhance public health surveillance in Yukon: a lower-resourced and remote setting. BMC Public Health.

[8] Chapman WW, Dowling JN, Baer A, Buckeridge DL, Cochrane D, Conway MA (2010). Developing syndrome definitions based on consensus and current use. J Am Med Inform Assoc.

[9] Jorm AF (2015). Using the Delphi expert consensus method in mental health research. Aust N Z J Psychiatry.

[10] Bekkering GE, Zeeuws D, Lenaerts E, Pas L, Verstuyf G, Matthys F (2016). Development and validation of quality indicators on continuing care for patients with AUD: a Delphi study. Alcohol Alcohol.

[11] Li Y, Ehiri J, Hu D, Zhang Y, Wang Q, Zhang S (2014). Framework of behavioral indicators for outcome evaluation of TB health promotion: a Delphi study of TB suspects and Tb patients. BMC Infect Dis.

[12] Debin M, Souty C, Turbelin C, Blanchon T, Boëlle PY, Hanslik T (2013). Determination of French influenza outbreaks periods between 1985 and 2011 through a web-based Delphi method. BMC Med Inform Decis Mak.

[13] Bourree F, Michel P, Salmi LR (2008). Consensus methods: review of original methods and their main alternatives used in public health. Rev Epidemiol Sante Publique.

[14] Aide au codage 2022 [updated Janvier 2022]. Available from: https://www.aideaucodage.fr/cim. Accessed 4 Aug 2022.

[15] International classification of diseases, tenth revision, clinical modification (ICD-10-CM): Centers for Disease Control and Prevention; 2021. Available from: https://www.cdc.gov/nchs/icd/icd10cm.htm. Accessed 4 Aug 2022.

[16] Smith GE, Elliot AJ, Lake I, Edeghere O, Morbey R, Catchpole M (2019). Syndromic surveillance: two decades experience of sustainable systems - its people not just data!. Epidemiol Infect.

[17] Burkom H, Loschen W, Wojcik R, Holtry R, Punjabi M, Siwek M (2021). Electronic surveillance system for the early notification of community-based epidemics (ESSENCE): overview, components, and public health applications. JMIR Public Health Surveill.

[18] WHO (2022). End-to-end integration of SARS-CoV-2 and influenza sentinel surveillance: revised interim guidance.

[19] Dinh MM, Berendsen Russell S, Bein KJ (2019). Diagnoses, damned diagnoses and statistics: dealing with disparate diagnostic coding systems within the New South Wales emergency department data collection. Emerg Med Australas.

[20] Morbey R, Hughes H, Smith G, Challen K, Hughes TC, Elliot AJ (2019). Potential added value of the new emergency care dataset to ED-based public health surveillance in England: an initial concept analysis. Emerg Med J.

[21] Bloom BM, Pott J, Thomas S, Gaunt DR, Hughes TC (2021). Usability of electronic health record systems in UK EDs. Emerg Med J.

[22] Faculty of Clinical Informatics. Consultation on a diagnostic code set for Same Day Emergency Care. 2021. https://facultyofclinicalinformatics.org.uk/same-day-emergency-care. Accessed 9 Sept 2022.

