# Supplementary figures and images for: Expert consultation using the on-line Delphi method for the revision of syndromic groups compiled from emergency data (SOS Médecins and OSCOUR®) in France
Source: BMC Public Health. 2022 Sep 21;22:1791. doi: 10.1186/s12889-022-14157-x (PMC9494916; doi:10.1186/s12889-022-14157-x)

**Additional file 1: Screen shot of the OSCOUR® survey questionnaire for Injury SG in the 2nd round**


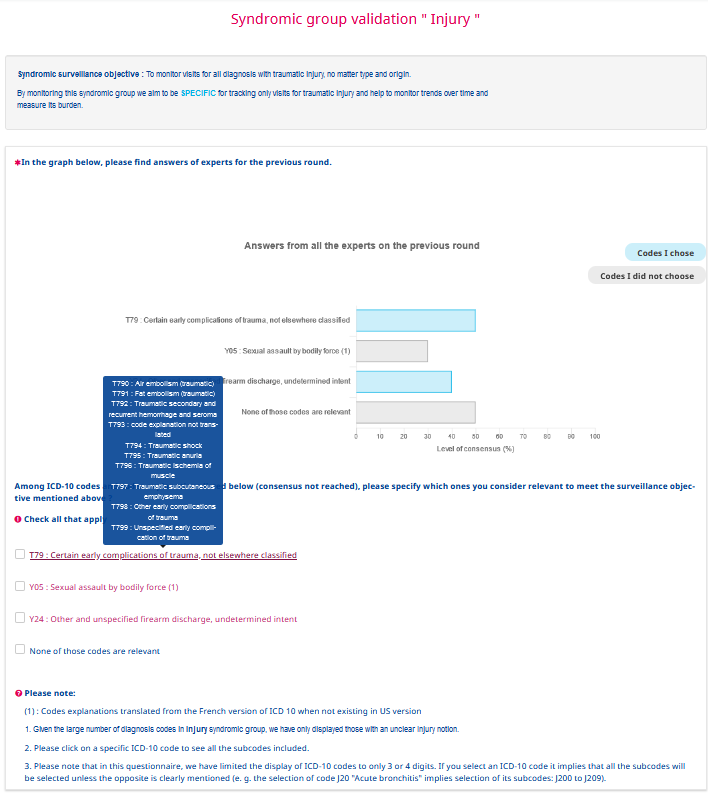

Supplement: Supplementary file 1 — Additional file 1. Screenshot of the OSCOUR® survey questionnaire for Injury SG in the 2nd round. This figure shows the display of the questionnaire for the syndromic group “injury” in the 2nd round of the Delphi OSCOUR® survey. On this screenshot, we have at the top the remind of surveillance objective. On the graph, each bar represents one diagnostic code, the proportion of consensus is represented on the abcissa axis. The blue color on the bar indicates to the participant the code he chose while the grey color indicates the codes he did not choose. Below the graph are listed the ICD-10 codes of syndromic group “injury”, the participants were invited to indicate again relevant diagnostic codes according to the surveillance objective. By rolling over T79 with the mouse, they could view included subcodes in the dark blue box. [file 12889_2022_14157_MOESM1_ESM.docx]
