# Supplementary material for: Expert consultation using the on-line Delphi method for the revision of syndromic groups compiled from emergency data (SOS Médecins and OSCOUR®) in France
Source: BMC Public Health. 2022 Sep 21;22:1791. doi: 10.1186/s12889-022-14157-x (PMC9494916; doi:10.1186/s12889-022-14157-x)
Supplement: Supplementary file 2 — Additional file 2. a. Number of participants contacted and having answered the Delphi SOS Médecins survey. The first column indicates the different rounds of the Delphi SOS Médecins survey. The next 3 columns indicate results for the different groups of the survey. The last column shows the total for all the three groups. The 3 first lines give the number of people per group to whom the questionnaire was sent for each round. The last 3 rows show the number of people who responded to the questionnaire and the participation rate in percentage (number of persons who completed the questionnaire by the number of persons contacted) in each group, for each round. b. Number of participants contacted and having answered the Delphi OSCOUR® survey. The first column indicates the different rounds of the Delphi OSCOUR® survey. The next 3 columns indicate results for the different groups of the survey. The last column shows the total for all the three groups. The three first line give the number of people per group to whom the questionnaire was sent for each round. The last 3 rows show the number of people who responded to the questionnaire and the participation rate in percentage (number of persons who completed the questionnaire by the number of persons contacted) in each group, for each round. [file 12889_2022_14157_MOESM2_ESM.zip › Additional file 2b.docx]

**Additional file 2b: Number of participants contacted and having answered the Delphi OSCOUR® survey**

|  | Group 1 | Group 2 | Group 3 | Group 4 | All groups |
| --- | --- | --- | --- | --- | --- |
|  | Number of persons contacted at each round (n) | | | | |
| Round 1 | 18 | 17 | 18 | 19 | 72 |
| Round 2 | 11 | 12 | 11 | 13 | 47 |
| Round 3 | 10 | 9 | 9 | 12 | 40 |
|  | Number of persons who completed the questionnaire (n) and participation rate (%) at each round | | | | |
| Round 1 | 11 (61%) | 12 (71%) | 11 (61%) | 13 (68%) | 47 (65%) |
| Round 2 | 10 (91%) | 9 (75%) | 9 (82%) | 12 (92%) | 40 (85%) |
| Round 3 | 5 (50%) | 8 (89%) | 9 (100%) | 12 (100%) | 34 (85%) |
