# Supplementary material for: Expert consultation using the on-line Delphi method for the revision of syndromic groups compiled from emergency data (SOS Médecins and OSCOUR®) in France
Source: BMC Public Health. 2022 Sep 21;22:1791. doi: 10.1186/s12889-022-14157-x (PMC9494916; doi:10.1186/s12889-022-14157-x)
Supplement: Supplementary file 3 — Additional file 3. a. Diagnostic codes by syndromic groups (SG) (n = 14) and their proportion of consensus in the 3 rounds of the Delphi SOS Médecins survey. The first column indicates the syndromic group, the 2nd column the surveillance objective and the 3rd the label of diagnostic codes. Proportions of consensus are indicated in column 4th to 6th. And the last column indicates if the diagnosis was kept or no in the syndromic group. b. Diagnostic codes by syndromic groups (SG) (n = 11) and their proportion of consensus in the 3 rounds of the Delphi OSCOUR® survey. The first column indicates the syndromic group, the 2nd column the surveillance objective and the 3rd the label of diagnostic codes. Proportions of consensus are indicated in column 4th to 6th. The last two columns indicate if the diagnosis was kept or no in the syndromic group and the number of subcodes. [file 12889_2022_14157_MOESM3_ESM.zip › Additional file 3a.docx]

**Additional file 3a: Diagnostic codes by syndromic group (SG) (n=14) and their proportion of consensus in the 3 rounds of the Delphi SOS Médecins survey**

|  |  |  | **Proportion of consensus (%)** | | |  |
| --- | --- | --- | --- | --- | --- | --- |
| **SG** | **Surveillance objective** | **Diagnostic code** | **1st round** | **2nd round** | **3nd round** | **Diagnostic code to be kept in the SG** |
| Meningitis | To monitor visits for meningeal syndrome or suspected meningitis regardless of the aetiology. By monitoring this SG we aim to be **specific** for tracking only visits for meningeal syndrome or suspected meningitis, and help to monitor trends over time and measure its burden. | Meningeal syndrome/Meningitis | 88% | - | - | Yes |
| Bronchiolitis | To monitor visits for bronchiolitis. By monitoring this SG we aim to be **specific** for tracking only visits for bronchiolitis and help to monitor trends over time and measure its burden. | Bronchiolitis | 100% | - | - | Yes |
| Influenza or influenza-like illness | To monitor visits for influenza or influenza-like illness. By monitoring this SG we aim to be **specific** for tracking only visits for Influenza or influenza-like illness and help to monitor trends over time and measure its burden. | Influenza or influenza-like illnes | 100% | - | - | Yes |
| Gastroenteritis | To monitor visits for gastroenteritis. By monitoring this SG we aim to be **specific** for tracking only visits for gastroenteritis and help to monitor trends over time and measure its burden. | Gastroenteritis | 100% | - | - | Yes |
| Vomiting | To monitor visits for isolated vomiting, no matter type and etiology. By monitoring this SG, we aim to be **sensitive** in order to early detect a maximum number of visits for isolated vomiting, with evocative symptoms or confirmed diagnosis by clinical or complementary medical examinations. | Vomiting | 100% | - | - | Yes |
| Diarrhea | To monitor visits for isolated diarrhea, regardless of etiology (including bloody diarrhea), with fever or not. By monitoring this SG, we aim to be **sensitive** in order to early detect a maximum number of visits for isolated diarrhea (including bloody diarrhea), with evocative symptoms or confirmed diagnosis by clinical or complementary medical examinations. | Diarrhea | 100% | - | - | Yes |
|  |  | Bloody diarrhea | 88% | - | - | Yes |
| Acute abdominal pain | To monitor visits for acute abdominal or pelvic pain, with no etiology identified, excepted urological and gynaecologic pain. By monitoring this SG, we aim to be **sensitive** in order to early detect a maximum number of visits for acute abdominal or pelvic pain (excepted urological and gynaecologic pain), without a specific pathology identified. | Acute abdominal pain | - | 100% | - | Yes |
|  |  | Colic | - | 88% | - | Yes |
|  |  | Cholecystitis | - | 88% | - | Yes |
|  |  | Newborn baby colic | - | 88% | - | Yes |
|  |  | Spasmodic colitis | - | 88% | - | Yes |
| Acute confusion | To monitor visits for acute confusion without an underlying pathology, regardless of etiology. By monitoring this SG, we aim to be **sensitive** in order to early detect a maximum number of visits for acute  confusion, with evocative symptoms or confirmed diagnosis by clinical or complementary medical examinations. | Confusional syndrome | 100% | - | - | Yes |
|  |  | Delirious state | 88% | - | - | Yes |
|  |  | Behavioural disorder* | - | 88% | - | Yes |
| Burns and corrosions | To monitor visits for burns and corrosions, regardless of etiology, excepted heatstroke. By monitoring this SG, we aim to be **sensitive** in order to early detect a maximum number of visits for burns and corrosions, with evocative symptoms or confirmed diagnosis by clinical or complementary medical examinations. | Burn | 100% | - | - | Yes |
| Hyperthermia and heat stroke | To monitor visits for hyperthermia or heatstroke or sunstroke, related to an external factor (high external temperature, sun). By monitoring this SG, we aim to be **sensitive** in order to early detect a maximum number of visits for hyperthermia or heatstroke. | Heat stroke | 100% | - | - | Yes |
|  |  | Dehydration* | - | 88% | - | Yes |
| Conjunctivitis | To monitor visits for conjunctivitis of any origin. By monitoring this SG, we aim to be **sensitive** in order to early detect a maximum number of visits for conjunctivitis, with evocative symptoms or confirmed diagnosis by clinical or complementary medical examinations. | Infectious conjunctivitis | 100% | - | - | Yes |
|  |  | Allergic conjunctivitis* | - | 100% | - | Yes |
|  |  | Miscellaneous Ophthalmology* | - | 13% | - | No |
| Injury | To monitor visits for all diagnosis with traumatic injury, no matter type and origin. By monitoring this SG, we aim to be **specific** for tracking only visits for traumatic injury and help to monitor trends over time and measure its burden. | Other fracture | 100% | - | - | Yes |
|  |  | Sprain | 100% | - | - | Yes |
|  |  | Miscellaneous injury | 88% | - | - | Yes |
|  |  | Cranial trauma without initial loss of consciousness | 88% | - | - | Yes |
|  |  | Painful pronation | 100% | - | - | Yes |
|  |  | Cranial trauma with initial loss of consciousness | 100% | - | - | Yes |
|  |  | Sutured wound | 100% | - | - | Yes |
|  |  | Wound | 100% | - | - | Yes |
|  |  | Dislocation | 88% | - | - | Yes |
|  |  | Fracture of the upper limb | 88% | - | - | Yes |
|  |  | Fracture of the lower limb | 88% | - | - | Yes |
|  |  | Rib fracture | 88% | - | - | Yes |
|  |  | Femoral neck fracture | 88% | - | - | Yes |
|  |  | Eye injury | 100% | - | - | Yes |
|  |  | Foreign body eye | 88% | - | - | Yes |
|  |  | Arc-eye | 88% | - | - | Yes |
|  |  | Contusion* | - | 100% | - | Yes |
|  |  | Burn* | - | 88% | - | Yes |
|  |  | Wound cross-infected | 75% | 63% | 88% | Yes |
|  |  | Animal bite-scratch* | - | 75% | 88% | Yes |
| Scabies and other ectoparasites | To monitor visits for scabies and other ectoparasites. By monitoring this SG, we aim to be **specific** for tracking only visits for scabies and other ectoparasites, and help to monitor trends over time and measure its burden. | Scabies | 100% | - | - | Yes |
| Urinary tract infection | To monitor visits for urinary tract infection of any origin. By monitoring this SG, we aim to be **specific** for tracking only visits for urinary tract infection, and help to monitor trends over time and measure its burden. | Cystitis | 100% | - | - | Yes |
|  |  | Pyelonephritis | 100% | - | - | Yes |
|  |  | Prostatitis* | - | 88% | - | Yes |

*Codes suggested by participants
