# Supplementary material for: Expert consultation using the on-line Delphi method for the revision of syndromic groups compiled from emergency data (SOS Médecins and OSCOUR®) in France
Source: BMC Public Health. 2022 Sep 21;22:1791. doi: 10.1186/s12889-022-14157-x (PMC9494916; doi:10.1186/s12889-022-14157-x)
Supplement: Supplementary file 3 — Additional file 3. a. Diagnostic codes by syndromic groups (SG) (n = 14) and their proportion of consensus in the 3 rounds of the Delphi SOS Médecins survey. The first column indicates the syndromic group, the 2nd column the surveillance objective and the 3rd the label of diagnostic codes. Proportions of consensus are indicated in column 4th to 6th. And the last column indicates if the diagnosis was kept or no in the syndromic group. b. Diagnostic codes by syndromic groups (SG) (n = 11) and their proportion of consensus in the 3 rounds of the Delphi OSCOUR® survey. The first column indicates the syndromic group, the 2nd column the surveillance objective and the 3rd the label of diagnostic codes. Proportions of consensus are indicated in column 4th to 6th. The last two columns indicate if the diagnosis was kept or no in the syndromic group and the number of subcodes. [file 12889_2022_14157_MOESM3_ESM.zip › Additional file 3b.docx]

**Additional file 3b: Diagnostic codes by syndromic group (SG) (n=11) and their proportion of consensus in the 3 rounds of the Delphi OSCOUR® survey**

|  |  |  | **Proportion of consensus (%)** |  |  |  |  |
| --- | --- | --- | --- | --- | --- | --- | --- |
| **SG** | **Surveillance objective** | **Diagnostic code** | **1st round** | **2nd round** | **3rd round** | **Diagnostic code to be kept in the SG** | **Number of subcodes (n)** |
| Toxic effect of animals | To monitor visits for toxic effect due to a contact with an animal excepted arthropods. By monitoring this SG, we aim to be **sensitive** in order to early detect a maximum number of visits for toxic effect due to a contact with an animal, at a symptomatic stage or with a diagnosis confirmed by clinical or complementary medical examinations. | X29: Contact with venomous animals or venomous plants with no additional information | 82% | - | - | Yes | 10 |
|  |  | T63: Toxic effect of contact with a venomous animal | 100% | - | - | Yes | 6 |
|  |  | X20: Contact with snakes and venomous lizards | 55% | 90% | - | Yes | 10 |
|  |  | X26: Contact with venomous marine animals and venomous marine plants | 64% | 90% | - | Yes | 10 |
|  |  | X27: Contact with other specified venomous animals | 64% | 90% | - | Yes | 10 |
| Drowning | To monitor visits for drowning, no matter origin. By monitoring this SG we aim to be **specific** for tracking only visits for drowning and help to monitor trends over time and measure its burden. | T751: Unspecified effects of drowning and nonfatal submersion | 92% | - | - | Yes | 0 |
|  |  | W65: Accidental drowning and submersion while in bath-tub | 92% | - | - | Yes | 10 |
|  |  | W66: Drowning and submersion following a fall in bath-tub | 83% | - | - | Yes | 10 |
|  |  | W67: Accidental drowning and submersion while in swimming-pool | 100% | - | - | Yes | 10 |
|  |  | W68: Drowning and submersion following a fall in swimming-pool | 92% | - | - | Yes | 10 |
|  |  | W69: Accidental drowning and submersion while in natural water | 100% | - | - | Yes | 10 |
|  |  | W70: Drowning and submersion following a fall in natural water | 83% | - | - | Yes | 10 |
|  |  | W73: Other specified cause of accidental non-transport drowning and submersion | 92% | - | - | Yes | 10 |
|  |  | W74: Unspecified cause of accidental drowning and submersion | 92% | - | - | Yes | 10 |
|  |  | V90: Drowning and submersion due to accident to watercraft | 92% | - | - | Yes | 10 |
|  |  | Y21: Drowning and submersion, undetermined intent | 92% | - | - | Yes | 10 |
|  |  | J81: Pulmonary oedema * | - | 11% | - | No | 0 |
| Seizures | To monitor visits for seizure, regardless of etiology. By monitoring this SG, we aim to be **sensitive** in order to early detect a maximum number of visits for seizure, with evocative symptoms or confirmed diagnosis by clinical or complementary medical examinations. | R56: Convulsions, not elsewhere classified | 100% | - | - | Yes | 2 |
|  |  | F10.06: Epileptic seizure related to an acute intoxication by alcohol | 92% | - | - | Yes | 0 |
|  |  | F10.31: Epileptic seizure related to an alcohol withdrawal | 92% | - | - | Yes | 0 |
|  |  | G40 : Epilepsy and recurrent seizures | 92% | - | - | Yes | 10 |
|  |  | G41: Status epilepticus | 100% | - | - | Yes | 5 |
|  |  | F11.06: Acute poisoning by opioid with convulsion | 75% | 89% | - | Yes | 0 |
|  |  | P90: Convulsions of newborn * | - | 100% | - | Yes | 0 |
|  |  | F1041: Delirium Tremens with seizure during alcohol withdrawal* | - | 89% | - | Yes | 0 |
|  |  | F1131: Opioid related mental and behaviour disorders, withdrawal syndrome, with convulsion* | - | 89% | - | Yes | 0 |
|  |  | F1141: Opioid related mental and behaviour disorders, withdrawal syndrome with delirium, with convulsion* | - | 89% | - | Yes | 0 |
|  |  | F1206: Cannabis related mental and behaviour disorders, acute intoxication, with convulsion* | - | 89% | - | Yes | 0 |
|  |  | F1231: Cannabis related mental and behaviour disorders, withdrawal syndrome, with convulsion* | - | 89% | - | Yes | 0 |
|  |  | F1241: Cannabis related mental and behaviour disorders, withdrawal syndrome with delirium, with convulsion* | - | 89% | - | Yes | 0 |
|  |  | F1306: Sedative or hypnotic related mental and behaviour disorders, acute intoxication, with convulsions* | - | 89% | - | Yes | 0 |
|  |  | F1331: Sedative or hypnotic related mental and behaviour disorders, withdrawal syndrome, with convulsion* | - | 89% | - | Yes | 0 |
|  |  | F1341: Sedative or hypnotic related mental and behaviour disorders, withdrawal syndrome with delirium, with convulsion* | - | 89% | - | Yes | 0 |
|  |  | F1406: Cocaine related mental and behaviour disorders, acute intoxication, with convulsion* | - | 89% | - | Yes | 0 |
|  |  | F1431: Cocaine related mental and behaviour disorders, withdrawal syndrome, with convulsion* | - | 89% | - | Yes | 0 |
|  |  | F1441: Cocaine related mental and behaviour disorders, withdrawal syndrome with delirium, with convulsion* | - | 89% | - | Yes | 0 |
|  |  | F1506: Mental and behaviour disorders due to the use of other stimulant including caffeine, acute intoxication, with convulsion* | - | 89% | - | Yes | 0 |
|  |  | F1531: Mental and behaviour disorders due to the use of other stimulant including caffeine, withdrawal syndrome, with convulsion* | - | 89% | - | Yes | 0 |
|  |  | F1541: Mental and behaviour disorders due to the use of other stimulant including caffeine, withdrawal syndrome with delirium, with convulsion* | - | 89% | - | Yes | 0 |
|  |  | F1606: Mental and behaviour disorders due to an acute intoxication to hallucinogen, with convulsion* | - | 89% | - | Yes | 0 |
|  |  | F1631: Hallucinogen related mental and behaviour disorders, withdrawal syndrome, with convulsion* | - | 89% | - | Yes | 0 |
|  |  | F1641: Hallucinogen related mental and behaviour disorders, withdrawal syndrome with delirium, with convulsion* | - | 89% | - | Yes | 0 |
|  |  | F1706: Tobacco related mental and behaviour disorders, acute intoxication, with convulsion * | - | 89% | - | Yes | 0 |
|  |  | F1731: Tobacco related mental and behaviour disorders, withdrawal syndrome, with convulsion* | - | 89% | - | Yes | 0 |
|  |  | F1741: Tobacco related mental and behaviour disorders, withdrawal syndrome with delirium, with convulsion * | - | 89% | - | Yes | 0 |
|  |  | F1806: Mental and behaviour disorders due to an acute intoxication to volatile solvents, with convulsion* | - | 89% | - | Yes | 0 |
|  |  | F1831: Volatile solvents related mental and behaviour disorders, withdrawal syndrome, with convulsion* | - | 89% | - | Yes | 0 |
|  |  | F1841: Volatile solvents related mental and behaviour disorders, withdrawal syndrome with delirium, with convulsion* | - | 89% | - | Yes | 0 |
|  |  | F1906: Mental and behaviour disorders due to multiple drugs et psycho-active substances, acute intoxication, with convulsion* | - | 89% | - | Yes | 0 |
|  |  | F1931: Withdrawal syndrome for multiple drugs et psycho-active substances, with convulsion* | - | 89% | - | Yes | 0 |
|  |  | F1941: Withdrawal syndrome for multiple drugs et psycho-active substances, with delirium, with convulsion* | - | 89% | - | Yes | 0 |
| Coma | To monitor visits for coma, regardless of etiology. By monitoring this SG, we aim to be **sensitive** in order to early detect a maximum number of visits for coma, with evocative symptoms or confirmed diagnosis by clinical or complementary medical examinations. | R40: Somnolence, stupor and coma | 100% | - | - | Yes | 2 |
|  |  | E100: Type 2 diabetes mellitus with hyperosmolarity | 83% | - | - | Yes | 0 |
|  |  | F1005: Alcoholic coma | 92% | - | - | Yes | 0 |
|  |  | E110: Non-insulin-dependent diabetes mellitus, with coma | 67% | 100% | - | Yes | 0 |
|  |  | E1100: Type 2 diabetes mellitus with hyperosmolarity without nonketotic hyperglycemic-hyperosmolar coma (NKHHC) | 67% | 100% | - | Yes | 0 |
|  |  | E1108: Type 2 diabetes (without insulin or unspecified) with coma | 67% | 100% | - | Yes | 0 |
|  |  | F1105: Opioid acute intoxication, with coma* | - | 89% | - | Yes | 0 |
|  |  | F1205: Cannabis related mental and behaviour disorders, acute intoxication, with coma* | - | 100% | - | Yes | 0 |
|  |  | F1305: Sedative or hypnotic related mental and behaviour disorders, acute intoxication, with coma* | - | 100% | - | Yes | 0 |
|  |  | F1405: Cocaine related mental and behaviour disorders, acute intoxication, with coma* | - | 100% | - | Yes | 0 |
|  |  | F1505: Mental and behaviour disorders due to the use of other stimulant including caffeine, acute intoxication, with coma* | - | 100% | - | Yes | 0 |
|  |  | F1605: Mental and behaviour disorders due to an acute intoxication to hallucinogen, with coma* | - | 100% | - | Yes | 0 |
|  |  | F1705: Tobacco related mental and behaviour disorders, acute intoxication, with coma* | - | 100% | - | Yes | 0 |
|  |  | F1805: Mental and behaviour disorders due to an acute intoxication to volatile solvents, with coma * | - | 100% | - | Yes | 0 |
|  |  | F1905: Mental and behaviour disorders due to multiple drugs et psycho-active substances, acute intoxication, with coma* | - | 100% | - | Yes | 0 |
|  |  | E870: Hyperosmolarity and hypernatremia* | - | 0% | - | No | 0 |
|  |  | E871: Hypo-osmolality and hyponatremia* | - | 0% | - | No | 0 |
|  |  | G934: Other and unspecified encephalopathy* | - | 22% | 13% | No | 0 |
| Cardiac rhythm and conduction disorders | To monitor visits for cardiac rhythm and conduction disorders regardless of etiology. By monitoring this SG, we aim to be **sensitive** in order to early detect a maximum number of visits for cardiac rhythm and conduction disorders, with evocative symptoms or confirmed diagnosis by clinical or complementary medical examinations. | I44: Atrioventricular and left bundle-branch block | 100% | - | - | Yes | 8 |
|  |  | I45: Other conduction disorders | 100% | - | - | Yes | 9 |
|  |  | I47: Paroxysmal tachycardia | 92% | - | - | Yes | 4 |
|  |  | I48: Atrial fibrillation and flutter | 100% | - | - | Yes | 6 |
|  |  | I49: Other cardiac arrhythmias | 100% | - | - | Yes | 8 |
|  |  | R00: Abnormalities of heart beat | 83% | - | - | Yes | 4 |
|  |  | I24: Other acute ischemic heart diseases* | - | 11% | - | No | 4 |
|  |  | I25: Chronic ischemic heart disease* | - | 11% | - | No | 9 |
| Chest pain | To monitor visit for chest pain, regardless of etiology. By monitoring this SG, we aim to be **sensitive** in order to early detect a maximum number of visits for chest pain, with evocative symptoms or confirmed diagnosis by clinical or complementary medical examinations. | R072: Precordial pain | 100% | - | - | Yes | 0 |
|  |  | R073: Other chest pain | 83% | - | - | Yes | 0 |
|  |  | R074: Chest pain, unspecified | 100% | - | - | Yes | 0 |
|  |  | R071: Respiratory chest pain | 67% | 89% | - | Yes | 0 |
|  |  | G580: Intercostal neuropathy* | - | 89% | - | Yes | 0 |
|  |  | I20: Angina pectoris * | - | 89% | - | Yes | 4 |
|  |  | R53: Fainting and fatigue * | - | 0% | - | No | 0 |
|  |  | I21: Acute myocardial infarction * | - | 78% | 88% | Yes | 6 |
|  |  | I22: Subsequent ST elevation (STEMI) and non-ST elevation (NSTEMI) myocardial infarction* | - | 78% | 88% | Yes | 4 |
|  |  | I26: Pulmonary embolism* | - | 78% | 88% | Yes | 2 |
|  |  | I71: Aortic aneurysm and dissection * | - | 78% | 88% | Yes | 1 |
| Asthma | To monitor visits for asthma attack. By monitoring this SG we aim to be **specific** for tracking only visits for asthma attack and help to monitor trends over time and measure its burden. | J45: Asthma | 91% | - | - | Yes | 4 |
|  |  | J46: Status asthmaticus | 100% | - | - | Yes | 0 |
| Bronchiolitis | To monitor visits for bronchiolitis. By monitoring this SG we aim to be **specific** for tracking only visits for bronchiolitis and help to monitor trends over time and measure its burden. | J21: Acute bronchiolitis | 100% | - | - | Yes | 3 |
| Acute bronchitis | To monitor visits for acute bronchitis, regardless of etiology. By monitoring this SG, we aim to be **sensitive** in order to early detect a maximum number of visits for acute bronchitis, with evocative symptoms or confirmed diagnosis by clinical or complementary medical examinations. | J20: Acute bronchitis | 100% | - |  | Yes | 10 |
|  |  | J40: Bronchitis, (not specified as acute or chronic) | 64% | 56% | 89% | Yes | 0 |
| Gastroenteritis | To monitor visits for gastroenteritis. By monitoring this SG we aim to be **specific** for tracking only visits for gastroenteritis. and help to monitor trends over time and measure its burden. | A09: Other gastroenteritis and colitis of infectious or unspecified origin | 85% | - | - | Yes | 3 |
|  |  | A08: Viral and other specified intestinal infections | 69% | 92% | - | Yes | 6 |
|  |  | K52: Other and unspecified noninfective gastroenteritis and colitis* | - | 25% | 17% | No | 5 |
| Hyponatraemia | To monitor visits for hyponatremia, regardless of etiology. By monitoring this SG, we aim to be **sensitive** in order to early detect a maximum number of visits for hyponatremia, with fainting or not, with evocative symptoms or confirmed diagnosis by clinical or complementary medical examinations. | E871: Hypoosmolarity and hyponatremia | 100% | - | - | Yes | 0 |
|  |  | E8710: Hyponatremia below 120 mmol/L | 77% | 92% | - | Yes | 0 |
|  |  | E8718: Other unspecified hypo-osmalarity and hyponatremia | 77% | 92% | - | Yes | 0 |
|  |  | E877: Fluid overload* | - | 8% | - | No | 0 |

*Codes suggested by participants
